# Supplementary material for: From sequence to enzyme mechanism using multi-label machine learning
Source: BMC Bioinformatics. 2014 May 19;15:150. doi: 10.1186/1471-2105-15-150 (PMC4229970; doi:10.1186/1471-2105-15-150)
Supplement: Additional file 2 — Java code of ml2db. Additional file ml2db_code.tar.gz contains the Java source code to run the multi-label machine learning experiments and save the results to database. The code’s Javadoc is included. [file 1471-2105-15-150-S2.zip › additional file 2/ml2db/ecmulan/doc/uk/ac/ed/inf/mulanxml/XmlCreatorManager.html]

XmlCreatorManager


JavaScript is disabled on your browser.


- Overview
- Package
- Class
- Use
- Tree
- Deprecated
- Index
- Help

- Prev Class
- Next Class

- Frames
- No Frames

- All Classes

- Summary:
- Nested |
- Field |
- Constr |
- Method

- Detail:
- Field |
- Constr |
- Method


uk.ac.ed.inf.mulanxml

## Class XmlCreatorManager

- java.lang.Object
- - uk.ac.ed.inf.mulanxml.XmlCreatorManager

- ---

    

  ```
  public class XmlCreatorManager
  extends java.lang.Object
  ```

  Checks the nature of the data: if the labels are EC numbers, it generates an
  ECNumber xml creator, if not, it generates a plain xml creator.

  Version:
  :   19 Jun 2012

  Author:
  :   Luna De Ferrari luna.deferrari@ed.ac.uk or
      luna.deferrari@st-andrews.ac.uk

- - ### Constructor Summary

    Constructors

    Constructor and Description

    `XmlCreatorManager(java.lang.String dbConnPath,
    java.lang.String query)`
  - ### Method Summary

    Methods

    | Modifier and Type | Method and Description |
    | --- | --- |
    | `LocalDbReader` | `getDbReader()` |
    | `java.util.TreeSet<java.lang.String>` | `getLabels()` |
    | `XmlCreator` | `getXmlCreator()` |

    - ### Methods inherited from class java.lang.Object

      `equals, getClass, hashCode, notify, notifyAll, toString, wait, wait, wait`

- - ### Constructor Detail


    - #### XmlCreatorManager

      ```
      public XmlCreatorManager(java.lang.String dbConnPath,
                       java.lang.String query)
      ```
  - ### Method Detail


    - #### getDbReader

      ```
      public LocalDbReader getDbReader()
      ```


    - #### getLabels

      ```
      public java.util.TreeSet<java.lang.String> getLabels()
      ```

      Returns:
      :   m\_labels


    - #### getXmlCreator

      ```
      public XmlCreator getXmlCreator()
      ```


- Overview
- Package
- Class
- Use
- Tree
- Deprecated
- Index
- Help

- Prev Class
- Next Class

- Frames
- No Frames

- All Classes

- Summary:
- Nested |
- Field |
- Constr |
- Method

- Detail:
- Field |
- Constr |
- Method
